# Clonal, Self-Renewing and Differentiating Human and Porcine Urothelial Cells, a Novel Stem Cell Population

**DOI:** 10.1371/journal.pone.0090006

**Published:** 2014-02-26

**Authors:** Hans M. Larsson, Francois Gorostidi, Jeffrey A. Hubbell, Yann Barrandon, Peter Frey

**Affiliations:** 1 Laboratory for Regenerative Medicine and Pharmacobiology, Institute for Bioengineering, School of Life Sciences and School of Engineering, Ecole Polytechnique Fédérale de Lausanne, Lausanne, Switzerland; 2 Laboratory of Experimental Surgery, Department of Experimental Surgery, Centre Hospitalier Universitaire Vaudois, Lausanne, Switzerland; 3 Laboratory of Stem Cell Dynamics, Institute for Bioengineering, School of Life Sciences, Ecole Polytechnique Fédérale de Lausanne, Lausanne, Switzerland; 4 Laboratory of Experimental Pediatric Urology, Department of Pediatric Urology, Centre Hospitalier Universitaire Vaudois, Lausanne, Switzerland; French Blood Institute, France

## Abstract

Although urothelial progenitor-like cells have been described in the human urinary tract, the existence of stem cells remains to be proven. Using a culture system that favors clonogenic epithelial cell growth, we evaluated and characterized clonal human urothelial cells. We isolated human urothelial cells that were clonogenic, capable of self-renewal and could develop into fully differentiated urothelium once re-implanted into the subcapsular space of nude mice. In addition to final urothelial cell differentiation, spontaneous formation of bladder-like microstructures was observed. By examining an epithelial stem cell signature marker, we found p63 to correlate with the self-renewal capacity of the isolated human urothelial clonal populations. Since a clinically relevant, long-term model for functional reconstitution of human cells does not exist, we sought to establish a culture method for porcine urothelial cells in a clinically relevant porcine model. We isolated cells from porcine ureter, urethra and bladder that were clonogenic and capable of self-renewal and differentiation into fully mature urothelium. In conclusion, we could isolate human and porcine cell populations, behaving as urothelial stem cells and showing clonogenicity, self-renewal and, once re-implanted, morphological differentiation.

## Introduction

Adult stem cells are currently used to treat patients with severe burns and hematological diseases [1], [2], [3]. To date, such adult stem cells displaying clonogenicity, self-renewal and differentiation capacity have not been characterized in human urothelium. Urothelial stem cells have been described in mice and were found to express sonic-hedgehog proteins in the basal cell layers of the bladder urothelium [4]. A more recent report has demonstrated that mouse urothelial stem cells are p63-positive as well [5]. This has not been shown in larger-animal models or humans, although the existence of human urothelial progenitor-like cells have been described in the human urinary tract by multiple groups [6], [7]. Clonogenic cell growth, however, ultimately proving the existence of human urothelial stem cells, has not been demonstrated *in vitro*. In larger-animal models, both porcine and bovine urothelial cells have been shown to be capable of forming colonies and to differentiate *in vitro* [8], [9].

To establish whether the human urinary tract possesses a stem cell capable of clonogenicity, self-renewal and differentiation *in vivo*, we used an established culture system for human epithelial stem cells: the 3T3-J2 culture system [10]. In parallel, we also explored porcine urothelial cells from different anatomical locations of the urinary tract, to have a clinically relevant animal model for investigations in urinary tract repair intended for human patients. Our criteria for human and porcine urothelial stem cells were clonogenicity, self-renewal *in vitro* and full urothelium differentiation capacities *in vivo*.

## Materials and Methods

### Ethical Human and Animal Research Approval

Ethical approval for working with human biopsies was given by the ethical board of the “Centre Hospitalier Universitaire Vaudois” (CHUV, Lausanne, CH). Furthermore, urinary tract biopsies were harvested following signed consent by the patients or their guardians. The “Office Vétérinaire Cantonal”, Vaud, Switzerland, approved all animal procedures.

### 3T3-J2 Cell Culture

Human and porcine urothelial cells were cultured on feeder layers of lethally irradiated 3T3-J2 cells as previously described by Rheinwald *et al*. [10]. 3T3-J2 fibroblasts in passage 4–12 were cultivated in Dulbecco-Vogt’s modification of Eagle’s medium (DMEM) (Life Technologies, CH) supplemented with 10% of fetal bovine serum (Life Technologies, CH). 3T3-J2 fibroblasts were grown in T162 flasks (Costar, USA) in a 10% CO_2_ atmosphere at 37°C 7 days after seeding, or when confluent. 3T3-J2 cells were dissociated with 0.05% trypsin and 0.01% EDTA and re-seeded. One day prior to seeding of the epithelial cells, 3T3-J2 cells were lethally irradiated by gamma radiation (60 Gy dose, MDS Nordion Gammacell 3000 irradiator, Best Theratronics, UK) and seeded at a density of 2×10^4^ cells/cm^2^.

### Human Urothelial Cell Isolation and Culture

Human urothelial cells were isolated from ureteral biopsies from pediatric donors undergoing open surgeries for non-malignant congenital anomalies (Table S1A). Primary human urothelial cell isolation was carried out as previously described by Southgate *et al.* [6]. The urinary tract biopsies were incubated for 16 h at 4°C in HBSS buffer supplemented with 0.1% EDTA (Sigma, CH), 10 mM HEPES (Life Technologies, CH) and aprotinin (1X, Roche, CH). Using forceps, the tissue was mechanically scraped. Scraped tissue was incubated with 2 mL collagenase IV (100 U/mL, Sigma, CH) at 37°C for 20 min. Finally, cells were strained through a 100 µm cell-strainer (Falcon, BD, CH) before being seeded onto the lethally irradiated 3T3-J2 cells. Human urothelial cells were cultured in a 10% CO_2_ atmosphere at 37°C in cFAD medium, which consists of DMEM and Ham’s F12 (Life Technologies, CH) medium (v/v 3∶1), supplemented with 20% of fetal bovine serum (FBS) (Life Technologies, Australia), adenine (24.3 µg/mL, Merck, CH), insulin (5 µg/mL, Sigma, CH), 3,3,5-triiodo-L-thyronine (T3) (2×10^–9^ M, Sigma, CH), hydrocortisone (0.4 µg/mL, Sigma, CH), cholera toxin (1×10^–10^ M, Sigma, CH), and 1% penicillin/streptomycin (Life Technologies, CH). All urothelial cultures were fed cFAD-containing FBS originating from the same batch. The urothelial cells were fed with cFAD supplemented with Epidermal growth factor (EGF) (10 ng/mL, Roche, CH) every 4 days or when the medium became acidic.

### Porcine Urothelial Cell Isolation and Culture

Porcine urinary tract biopsies for cell isolation were kindly donated from the University of Bern Veterinarian School and the CHUV. GFP-porcine urothelial cells were isolated from urinary tract biopsies of a 12-month-old transgenic pig constitutively expressing eGFP bought from the Institute of Molecular Animal Breeding/Gene Center (LMU, Munich, Germany) [11]. Details of the porcine donors can be found elsewhere (Table S1). Bladder biopsies were taken from the bladder trigone and the bladder dome, while urethral biopsies were taken from the proximal urethra and ureteral biopsies were taken from mid distance between the ureteral meatus and the kidney. Porcine epithelial cells were isolated and cultured as previously described by Grasset *et al.* [12]. Biopsies were minced into small pieces (1–3 mm^2^) and incubated in a cell isolation solution (trypsin (0.02%), EDTA (0.1%) and collagenase A (1 mg/mL, Roche, CH)) at 37°C under gentle stirring. Every hour, the cell isolation solution was replaced. Each batch of recovered cell isolation media was centrifuged (1300 rpm, 5 min) and filtered through a 100 µm cell-strainer before being seeded into dishes or flasks containing lethally irradiated 3T3-J2 cells. Porcine epithelial cells were grown in the same culture condition as the human urothelial cells, except using 10% of the same batch of FBS.

During harvesting of porcine tissues for cell isolation, biopsies of native porcine tissues were taken and fixed in 10% natural buffered formalin (NBF 10%). Paraffin embedded native porcine tissues were later used as controls for immunohistochemistry. Porcine skin epithelial tissue was used as negative control in immunohistochemistry. Cultured porcine keratinocytes and thymus cells were used as negative controls in immunofluorescence.

### Mass-cultivation of Urothelial Cells

Mass-cultivated urothelial cells were seeded at a density of 5×10^4^–2×10^5^ cells into 30 mm, 60 mm or T25 flasks containing lethally irradiated 3T3-J2 cells. Cells were passaged every week by dissociating growing epithelial cells in 0.05% trypsin/0.1% EDTA and then by re-plating them at the appropriate density to achieve cell confluence within 7 days. Population doubling (PD) was calculated using the following formula: PD = Log(N/N0)/Log(2), where N0 is the number of seeded viable cells and N is the number of viable cells at the time of passage counted, using a hemocytometer.

### Colony-forming Assay

Each time a new urothelial cell population was passaged, a colony-forming assay was performed. The colony-forming assay consisted of seeding urothelial cells at a density of 50 to 5000 cells into duplicated 60 mm or 100 mm indicator culture dishes, containing lethally irradiated 3T3-J2 cells. The cells were cultivated for 12 days as described above, before being fixed in NBF 10% and stained with rhodamine-B (1%, Sigma, Germany). Colony-forming efficiencies (CFE) were calculated by dividing the number of colonies by the initial number of seeded cells in each plate.

### Clonal Analysis

**Figure 1:**
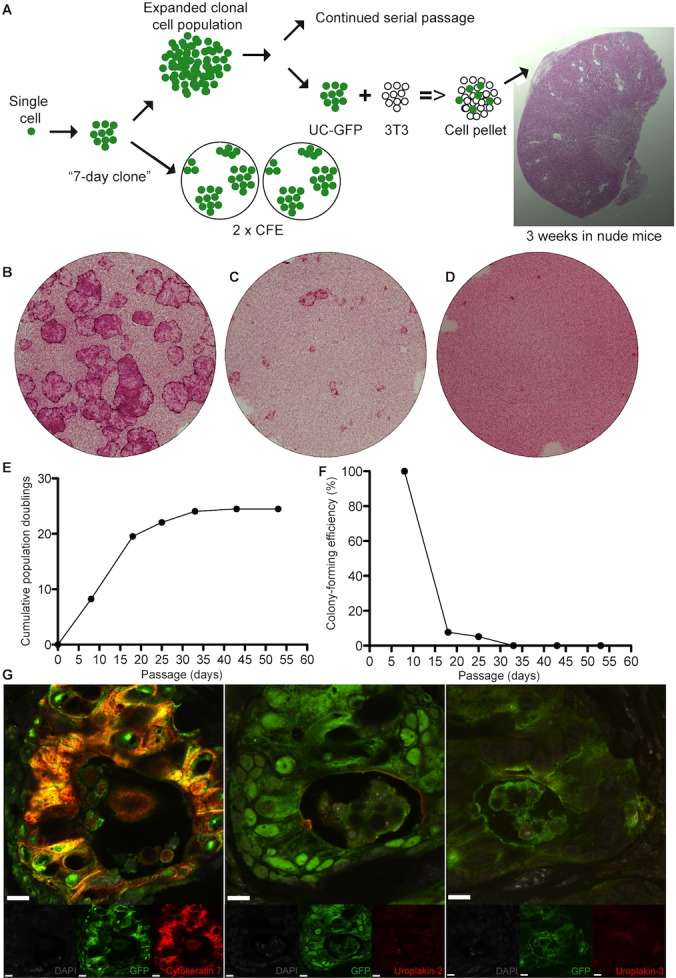
Human clonal urothelial cells arising from single ureteral cell. (A) Schematics showing the passage from the single selected cell to the in vivo implantated clonal cell pellet. (B,C and D) Human urothelial holoclone, meroclone and paraclone cultures arising from one single ureteral cell. (E and F) Growth curve of a human urothelial holoclone (G) *In vivo* urothelial differentiation of human ureteral urothelial holoclone pellets implanted into the subcapsular space of the Swiss nu/nu mice, expressing cytokeratin 7, uroplakin-2 and uroplakin 3 (scale bars, 10 µm). Note the “micro-bladder” like structure.

Single cells from passages 1–3 were aspirated into a Pasteur pipette under a Zeiss Axiovert inverted microscope (Germany) using a 10x objective and were subsequently inoculated into a tissue culture dish containing lethally irradiated 3T3-J2 cells. Small and round cells were selected following the rationale of Barrandon et al [13]. Cultures were fed every 3–4 days with cFAD medium supplemented with 10 ng/mL human recombinant EGF, as described above. After 7 days, adherent single cells that formed colonies were identified and imaged with an inverted microscope (Zeiss, Germany). Areas of 7-day clones were measured with the ImageJ software (Clonal Area = CA), while cell numbers in the 7-day colonies were counted manually (Estimated cell number = Estim CN). Population doubling for each 7-day clones (Generation number = GN) was calculated from using the same formula as previously described in the method section, but with N0 = 1 and N = Estim CN. Generation time was calculated dividing the time of culture (7 days = 168 h) with the generation number. Clones were detached from the dishes with 0.05% trypsin and 0.01% EDTA and re-seeded, 2 dishes were inoculated each with 1/7 of cell suspensions obtained (for CFE). The rest ( = 5/7 of cell suspensions) was inoculated for clonal population expansion (Figure 1A).

Human and porcine clones arising from epidermal single cells can be classified as holoclones, meroclones or paraclones depending on the clone’s capacity to form aborted colonies [12], [14]. We used the following similar criteria to classify aborted and growing urothelial colonies under a binocular microscope defining “Growing” as having a colony diameter of ≥2 mm, “Aborted” of <2 mm, and “Aborted” as having a highly irregular colony shape.

Clones that formed 0–5% aborted colonies were classified as urothelial holoclones. Conversely, if a clone formed 100% aborted colonies or no colonies, it was classified as an urothelial paraclone. Clones that formed more than 5% but fewer than 100% aborted colonies were classified as urothelial meroclones.

### Lentiviral Infection for Human Urothelial Cell GFP Transduction

Titers of lentivirus containing the hPGK-GFP lentivector were kindly donated from Professor D. Trono (EPFL, Switzerland). 1 µL of lentivirus (titer 5*10^9^ TU/mL) was applied to 75% confluent human urothelial cells cultured in 6 well plate at passage 1.

### Immunohistochemistry/Fluorescence

Biopsies were fixed in 10% NBF and embedded in paraffin. Sections were prepared at a thickness of 8 µm. Hematoxylin and eosin staining was performed on all biopsies. Primary antibodies used for immunohistochemistry/fluorescence were donkey anti- goat uroplakin-2 (1∶500 dilution, Labforce, CH), mouse anti-uroplakin-3 (1∶50 dilution, Progen, Germany), mouse anti-cytokeratin 7 (1∶1000 dilution, Abcam, CH), rabbit anti-GFP (1∶500 dilution, Life Technologies, CH), mouse anti-Ki67 (1∶100 dilution, BD Pharmingen, CH) and mouse anti-p63 (1∶100 dilution, Neomarker, US). Secondary antibodies used were Alexa-Fluor 488, Alexa-Fluor 568, or Alexa-Fluor 647 (Molecular Probes, Life Technologies, CH).

For immunofluorescence, cells were cultured on glass cover slips in 12-well plates and fixed in 10% NBF (20 min, at 4°C) after 8–12 days. Cells were permeabilized with 0.4% saponin (Applichem, CH) in D-PBS (Life Technologies, CH) for 30 min. After blocking for 1 h (3% BSA and 0.4% saponin in D-PBS), the cells were incubated with primary antibodies at room temperature for 2 h. After washing, the cells were incubated with secondary antibodies for 2 h. After multiple washing steps, Hoechst 33342 (Life Technologies, CH) was added to the cells and incubated for 10 min before imaging. Images were taken with a LSM 700 confocal laser-scanning microscope (Zeiss, Germany). For negative controls, the primary antibody was omitted.

### *In vivo* Nude Mice Experiments

The renal subcapsular space of Swiss nu/nu mice (Charles-River Breeding laboratories, France) was used as an ectopic location for implanted urothelial pelleted cells. The implanted urothelial pelleted cells were a mix of 2.5*10^5^ GFP positive urothelial cells plus 2.5*10^5^ non-lethally irradiated 3T3-J2 cells. After 3 wk, kidneys were harvested and imaged with a fluorescence stereomicroscope (Leica, Germany) to locate GFP positive cells. Kidneys were fixed in 10% NBF and embedded in paraffin for histological analysis.

The dorsal subdermal space of Swiss nu/nu mice was also used as an ectopic location for implanted urothelial sheets following the technique described in Barrandon *et al.* [15]. The implanted sheets consisted of 12 day cultured urothelial cells treated with Dispase-II (Roche, CH), initially seeded at a density of 1*10^5^ GFP positive urothelial cells. After 3 wk, the subdermal implant was harvested and imaged with a fluorescence stereomicroscope (Leica, Germany) to locate GFP positive cells. The tissue segments were fixed in 10% NBF and embedded in paraffin for histological analysis.

## Results

### Characterization of Mass-cultured Human and Porcine Urothelial Cells

We observed that both human and porcine urothelial cells arising from respective biopsies were capable of forming colonies in culture (Figure S1). Urothelial cells of human native ureters as well as native porcine bladder and ureter expressed uroplakin-2 and uroplakin-3 (Figure S2C, S3C, S4C and S5C). In contrast, the urothelial cells of native porcine urethral tissue only expressed uroplakin-2 but not uroplakin-3 (Figure S6C). We also found that human and porcine urothelial cells cultured *in vitro* for 8 days widely expressed a general marker of urothelial cells, cytokeratin-7, but only expressed uroplakin-2 spot-wise in a sparse manner (Figure S2D, S3D, S4D, S5D and S6D). However, none of the human or porcine urothelial cells cultured *in vitro* for this period expressed uroplakin-3 (Figure S2D, S3D, S4D, S5D and S6D).

**Figure 2:**
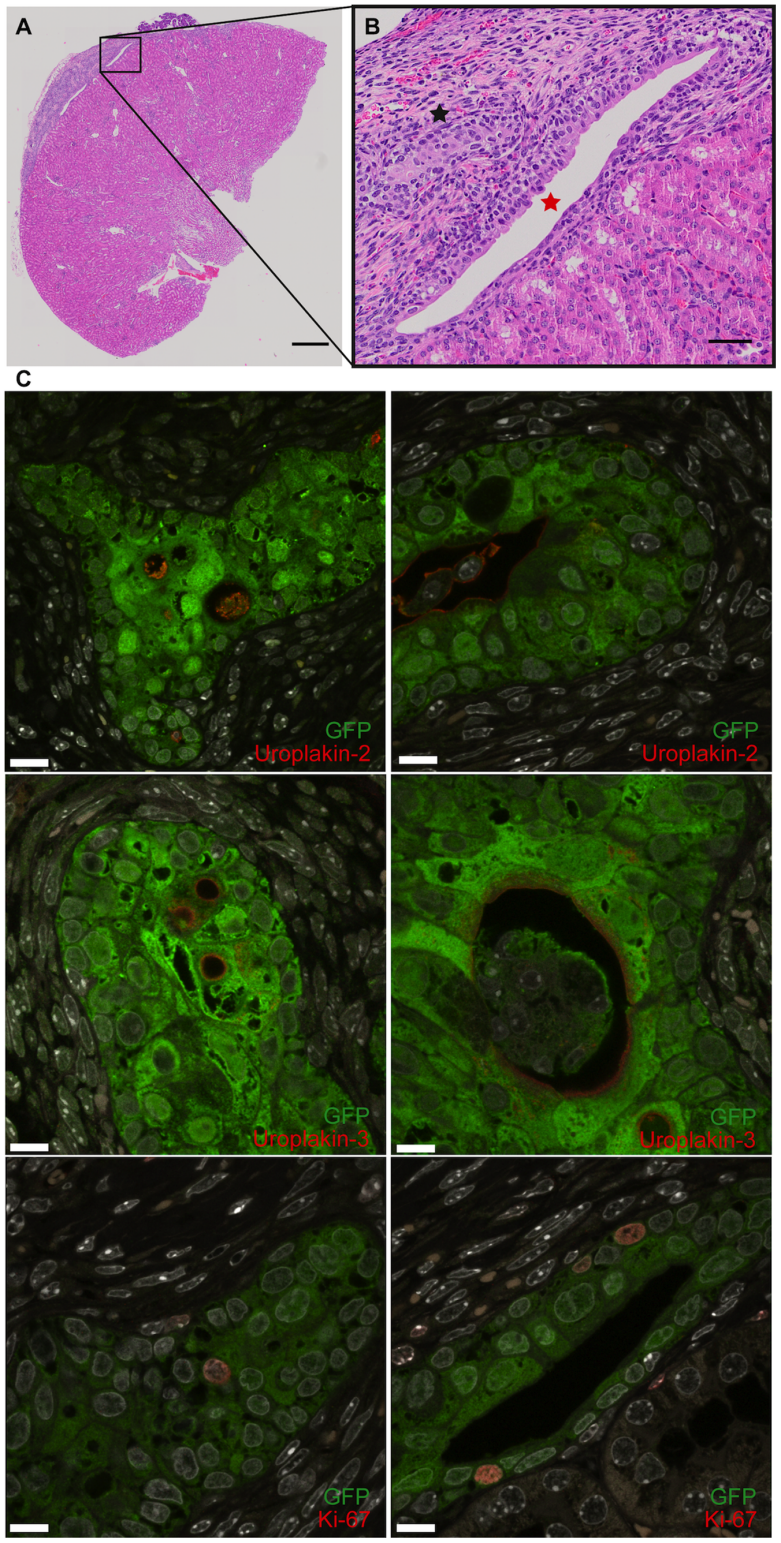
Urothelial cell differentiation and “micro-bladder” formation in mice. (A and B) Hematoxylin & eosin (H&E) staining of an implanted mass-cultured, native porcine bladder urothelial cell pellet into the subcapsular space of a Swiss nu/nu mice kidney (A: scale bar 500 µm, B: scale bar 50 µm, urothelial bundle-like structure indicated with black star and epithelial “micro-bladder” like structure indicated with red star). (C–H) Immunohistochemistry of the implanted urothelial pellet forming urothelial bundle-like structures (C, E and G) and “micro-bladder” like structures (D, F and H) using antibodies against uroplakin-2 (C and D), uroplakin-3 (E and F) and Ki-67 (G and H) (scale bars, 10 µm).

We sought to develop an *in vivo* model to study full differentiation of the urothelium. We tested two ectopic locations to implant GFP positive porcine mass-cultured urothelial cells in Swiss nu/nu mice. Dispase-treated sheets of urothelial cells cultured for 12 days were implanted into the dorsal sub-dermal space of the nude mice and were compared to urothelial cells implanted as a pellet under the kidney capsule. We sacrificed the animals after 3 wk and studied the expression of uroplakin-2 and uroplakin-3 in GFP positive cells. The urothelial sheets on the back of the mice formed a uniform sheet expressing uroplakin-2, but not uroplakin-3 (Figure S7). On the other hand the pellets implanted beneath the renal capsule formed urothelial bundle-like and urothelial “micro-bladder”-like structures with a lumen (Figure 2A and 2B). Both of these structures expressed uroplakin-2 and uroplakin-3 (Figure 2C). Furthermore we observed that they expressed a proliferation marker, Ki-67, suggesting that the GFP-urothelial cells were proliferating beneath the kidney capsule (Figure 2C).

We found that porcine ureteral, urethral, bladder dome and trigone cells grew well in the 3T3-J2 culture system, showing high colony-forming efficiencies for all the isolated biopsies (independent on age of donors) (Figure S3A–B, S4A–B, S5A–B and S6A–B). We did not observe any major growth differences between the different anatomical harvesting locations. Next, we investigated whether the porcine ureteral, urethral, bladder dome and trigone urothelial cells had similar differentiation capacities in the mouse kidney capsule model. We observed that the porcine ureteral, bladder dome and trigone cells formed fully differentiated urothelium, expressing uroplakin-2 and uroplakin-3 (Figure S3E, S4E and S5E). Interestingly, implanted urethral urothelial cells expressed uroplakin-2, following the protein expression pattern of native urethral tissue (Figure S6C and S6E), but as would be expected did not express uroplakin-3. Confirming the specificity of these antibodies for urothelium, we also found porcine skin biopsies as well as 8-day *in vitro* cultured porcine keratinocytes and thymus, to be negative for cytokeratin-7, uroplakin-2 and uroplakin-3 (Figure S8).

### Clonogenicity, Self-renewal in vitro and Urothelial Differentiation *in vivo* of Human Urothelial Cells Isolated from the Ureter

We explored whether handpicked cells with an elongated Pasteur pipette under an inverted microscope could initiate clonal growth. Following the rationale of Barrandon *et al.* [13], we selected small and round urothelial cells, and observed the development of clonal populations with different growth capacities. Applying the similar definitions for classifying the clonal type, based on aborted colony percentages as previously used for human epidermal, hair-follicle and corneal epithelial clonal cells [14], [16], [17], we observed that the selected clonal populations formed urothelial holoclones, meroclones and paraclones (Figure 1B, 1C and 1D) (Table 1).

**Table 1:** Clonal analysis of human ureteral cells.

Clone	CA day 7 (mm^2^)	Estim CN	GT (h)	GN	CFE (%)	GC (nb)	AB (nb)	AB (%)	Clonal type
1	1.295	1634	15.7	10.6	31.2	97	5	4.90	Holoclone
2	0.733	1209	16.4	10.2	19.9	46	2	4.17	Holoclone
3	0.243	330	20.1	8.36	3.04	0	2	100	Paraclone
4	1.378	1243	16.3	10.3	6.84	3	14	82.3	Meroclone
5	1.294	2661	14.8	11.4	4.51	12	12	50.0	Meroclone

(CA = clone area, Estim CN = estimated cell number, GT = generation time, GN = generation number, GC = growing colony, nb = number, AB = aborted).

**Figure 3:**
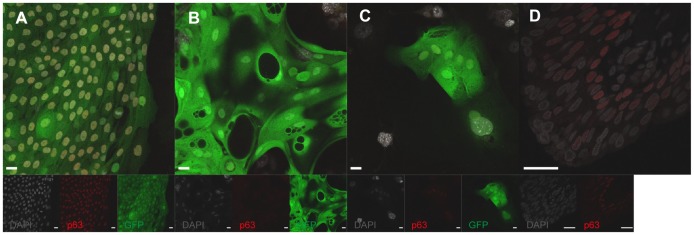
p63 expression in clonal human urothelial cells and urothelium. (A) p63-expression in a human urothelial holoclone culture at passage 2. (B) p63-expression in a human urothelial holoclone culture at passage 5. (C) p63-expression in a human urothelial meroclone culture at passage 2. (D) p63-expression in a human ureter biopsy (scale bars, 10 µm). Note the DAPI positive together with GFP negative cells are 3T3-J2 cells and DAPI positive together with GFP positive cells are urothelial cells.

The human urothelial holoclone could not be serially passaged longer than 25 population doublings with a decreased colony-forming efficiency over 45 days of culture, after which senescence occurred (Figure 1E and 1F). We observed that cells of human urothelial holoclones stained strongly and homogenously for p63 at an early passage (passage 2), while at later passage (passage 5) the urothelial holoclones expression of p63-expression was weak or not even present in colonies (Figure 3A and 3B). Evident from these images are the cell morphology differences going from a non-stretched cell morphology at passage 2 (characteristic of a proliferative urothelial phenotype) to a stretched cell morphology at passage 5 (characteristic of a differentiated/senescent urothelial cell phenotype) (Figure 3A and 3B). When we examined urothelial meroclones at an early passage (passage 2), we observed mixed p63-expression, ranging from strong, weak to no expression (Figure 3C). Interestingly, in human ureter biopsies we saw a strong p63-expression in the basal cells in the urothelium, a weak p63-expression in the intermediate cells and no p63 expression in the superficial cells (Figure 3D).

When we implanted the GFP-positive human urothelial holoclones in the kidney capsule model, we also found that these had the capacity to form “micro-bladder”-like structures, expressing uroplakin-2 and uroplakin-3, protein markers for fully differentiated urothelium (Figure 1G).

### Clonogenicity, Self-renewal in vitro and Urothelial Differentiation *in vivo* of Porcine Urothelial Cells Isolated from the Ureter, Bladder and Urethra

**Figure 4:**
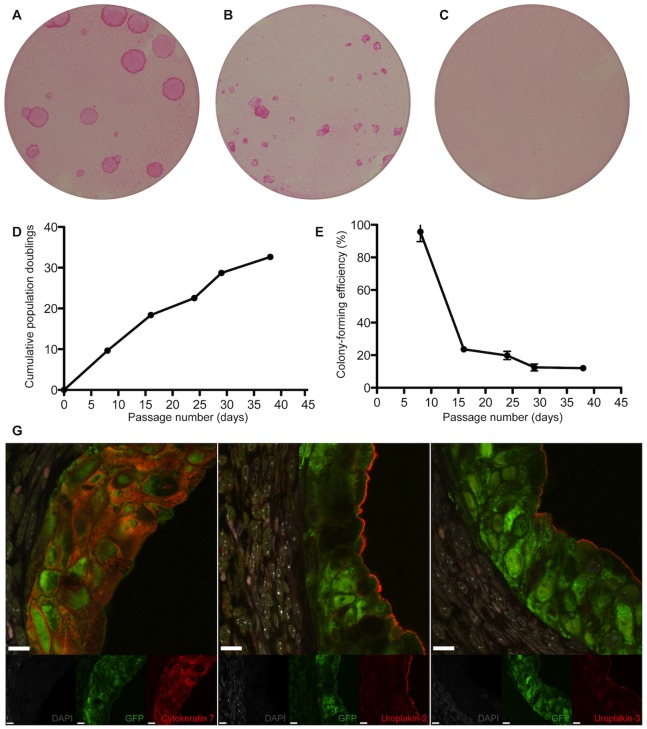
Porcine clonal urothelial cells arising from a single ureteral cell. (A, B and C) Porcine urothelial holoclone, meroclone and paraclone cultures arising from a single ureteral cell. (D and E) Growth curves of a porcine urothelial holoclone. (F) *In vivo* urothelial differentiation of porcine ureteral urothelial holoclone pellets implanted into the subcapsular space of the Swiss nu/nu mice, expressing cytokeratin 7, uroplakin-2 and uroplakin 3 (scale bars, 10 µm). Note the “micro-bladder” like structure.

**Figure 5:**
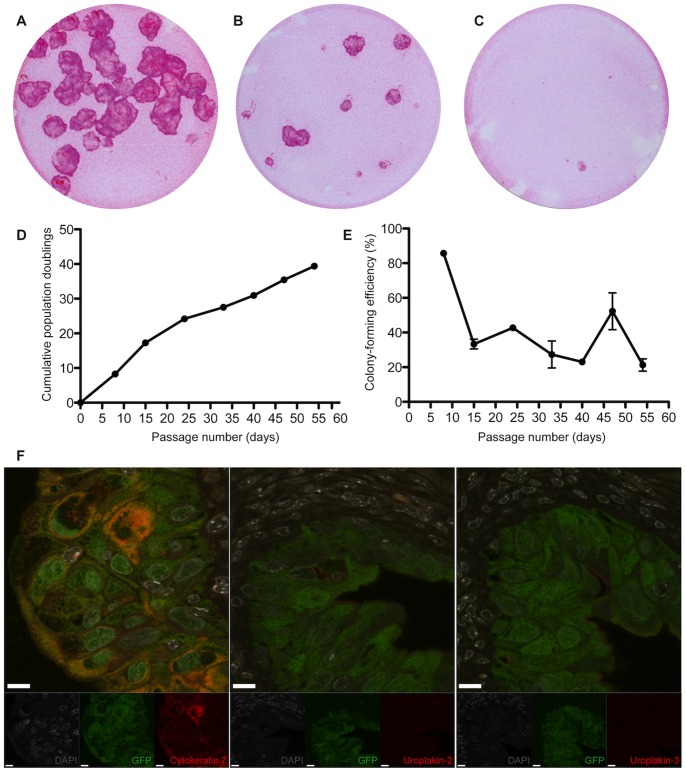
Porcine clonal urothelial cells arising from a single bladder cell. (A, B and C) Porcine urethelial holoclone, meroclone and paraclone cultures arising from a single bladder cell. (D and E) Growth curves of a porcine urothelial holoclone. (F) *In vivo* urothelial differentiation of porcine bladder urothelial holoclone pellets implanted into the subcapsular space of the Swiss nu/nu mice, expressing cytokeratin 7, uroplakin-2 and uroplakin 3 (scale bars, 10 µm). Note the “micro-bladder” like structure.

**Figure 6:**
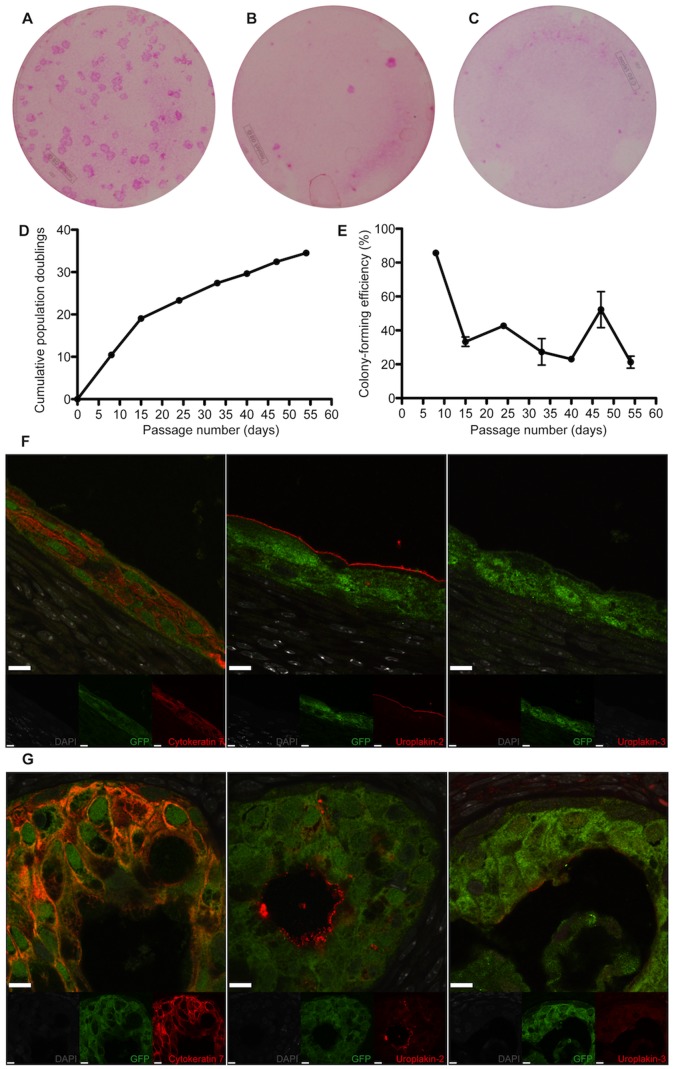
Porcine clonal urothelial cells arising from a single urethral cell. (A, B and C) Porcine urethelial holoclone, meroclone and paraclone cultures arising from a single urethral cell. (D and E) Growth curves of a porcine urothelial holoclone. (F, G) *In vivo* urothelial differentiation of porcine urethral urothelial holoclonal cell pellets implanted into the subcapsular space of the Swiss nu/nu mice, expressing cytokeratin 7, uroplakin-2 and uroplakin 3 (scale bars, 10 µm). Note the “micro-bladder” like structure.

Studying the *in vitro* behavior of cells harvested from the porcine bladder, ureter and urethra, we observed the same clonogenic capacity as the cells harvested from the human ureter. Porcine clonogenic urothelial cells were, as for the human cells, classified as urothelial holoclones, meroclones or paraclones, depending on the growth capacities. (Figure 4A–C, 5A–C and 6A–C) (Table 2, 3 and 4). Compared to human holoclones, porcine holoclones had a much greater growth capacity. It could be demonstrated that after 38 days of passaging, porcine urothelial holoclone showed 30 population doublings and thereafter were still in a growing phase (Figure 4D–E, 5D–E and 6D–E).

**Table 2:** Clonal analysis of porcine ureteral cells.

Clone	CA day 7 (mm^2^)	Estim CN	GT (h)	GN	CFE (%)	GC (nb)	AB (nb)	AB (%)	Clonal type
1	0.875	721	17.7	9.49	99.9	142	2	1.38	Holoclone
2	1.405	895	17.1	9.81	67.0	118	3	2.48	Holoclone
3	0.891	304	20.4	8.24	4.95	0	3	100	Paraclone
4	1.765	1310	16.2	10.4	39.3	54	49	47.6	Meroclone
5	0.284	639	18.0	9.32	33.7	21	22	51.2	Meroclone

(CA = clone area, Estim CN = estimated cell number, GT = generation time, GN = generation number, GC = growing colony, nb = number, AB = aborted).

**Table 3:** Clonal analysis of porcine bladder cells.

Clone	CA day 7 (mm^2^)	Estim CN	GT (h)	GN	CFE (%)	GC (nb)	AB (nb)	AB (%)	Clonal type
1	8.372	2500	14.9	11.3	56.0	272	8	2.86	Holoclone
2	0.098	185	22.3	7.53	81.1	29	1	3.33	Holoclone
3	0.009	12	46.9	3.58	83.3	0	2	100	Paraclone
4	0.035	90	25.9	6.49	55.5	8	2	20.0	Meroclone
5	0.155	140	23.6	7.13	39.3	8	3	27.3	Meroclone

(CA = clone area, Estim CN = estimated cell number, GT = generation time, GN = generation number, GC = growing colony, nb = number, AB = aborted).

**Table 4:** Clonal analysis of porcine urethral cells.

Clone	CA day 7 (mm^2^)	Estim CN	GT (h)	GN	CFE (%)	GC (nb)	AB (nb)	AB (%)	Clonal type
1	1.156	990	16.9	9.95	79.9	151	7	4.43	Holoclone
2	0.072	220	21.6	7.78	2.28	0	1	100	Paraclone
3	0.069	371	19.7	8.53	13.5	5	5	50.0	Meroclone
4	0.140	289	20.6	8.17	27.7	12	4	25.0	Meroclone

(CA = clone area, Estim CN = estimated cell number, GT = generation time, GN = generation number, GC = growing colony, nb = number, AB = aborted).

Next we analyzed the differentiation capacity of the porcine bladder-, porcine ureter-, and porcine urethra-holoclones in the kidney capsule model. We observed that all the implanted holoclones had the capacity to form “micro-bladder”-like structures. The porcine ureteral- and bladder-holoclones expressed both uroplakin-2 and uroplakin-3 (4F and 5F). Interestingly, in one of the three kidneys harvested from animals with implanted urethral holoclones, one micro-bladder was observed to express uroplakin-3 (Figure 6G), however, all other micro-bladders formed by the urethral holoclones only expressed uroplakin-2 and not uroplakin-3 (Figure 6F).

## Discussion

Human and porcine cell populations derived from a single urothelial cell, can self-renew *in vitro* and differentiate into mature urothelium *in vivo,* and therefore represent clonogenic urothelial stem cell populations. First, we aimed at reproducing the findings that urothelial cells had the capacity to form colonies at low cell seeding density when seeded using the 3T3-J2 culture system, as reported by Wu in the 1980’s [18]. These results encouraged us to perform clonal analysis of urothelial cells. The commonly applied urothelial cell culture technique uses a feeder cell-free culturing system [6], which does not allow clonal cell analysis. Using the 3T3-J2 culture system, we demonstrated that both human and porcine urothelial cells could initiate colony growth when seeded at low densities and clonal cell populations based on single cell seeding. However, the decrease of CFE, the premature change of morphology together with the loss of p63 expression indicates that the 3T3-J2 culture system can be improved for human urothelial stem cells expansion.

Further we evaluated if cultured urothelial cells can differentiate into a superficial urothelial cell type, representing the mature urothelial umbrella cells, being in contact with urine. In these cells, uroplakins are the main responsible molecules for the barrier function, forming protein plaques and preventing the urine from entering surrounding tissues. The uroplakin family consists of four proteins that make up these plaques, consisting of pairs of uroplakin-1a with uroplakin-2 and uroplakin-1b with uroplakin-3 [19]. We observed that after implantation of human and porcine urothelial cell pellets, generated from a single urothelial cells, into the renal subcapsular space of Swiss nu/nu mice, uroplakin-2 and uroplakin-3 expression were initiated. However, implanting urothelial sheets into the dorsal subdermal space of nude mice, only the expression of uroplakin-2 but not of uroplakin-3 was observed during the observation period of 3 weeks. It seems that urothelial cell pellets implanted into the renal subcapsular space favor differentiation into mature urothelial cells, making it a more suitable assay to study urothelial differentiation. We noticed that uroplakin-3 expression was only found after vacuole formation within urothelial bundle structures and at a later state within the “micro-bladder” like structures but not in the tight urothelial bundles. It can be speculated that an up-regulation of the PPARγ signal pathway and a down-regulation of EGF-pathway seen in *in vitro* cultured urothelial cells is due to the 3T3-J2 cells in the cell pellet [20]. We have not observed that rat or human thymic epithelial cells form these “micro-bladder” structures when implanted into the renal subcapsular space [21]. Indicating the “micro-bladder” like structures are specific for implanting urothelial cells. However, in literature it has been described that mouse embryonic stem cells are able to form similar “micro-bladder” like structures, when implanted together with micro dissected embryonic rat urogenital sinus into the renal subcapsular space [22]. Mouse bladder urothelial cells together with embryonic rat urogenital sinus also forms the “micro-bladder” like structures [23]. From these data it seems that “micro-bladder” like structures, can be formed either with 3T3-J2 cells or micro dissected embryonic rat urogenital sinus as carriers.

We aimed to establish whether there is a difference in porcine urothelial cell growth and differentiation capacities between different cell harvesting locations, as previously reported for bovine urothelial cells by Liang *et al.* [9]. This might become important if isolation of urothelial cells from a disease-free location of the urinary tract is performed to replace neoplastic or diseased tissues. From our porcine *in vitro* and *in vivo* data, we could not observe any difference in cell growth and differentiation between cells harvested from the ureter and the bladder. The *in vitro* cell growth and differentiation capacity of urothelial cells harvested from the urethra was comparable to the one of cells harvested from the bladder. However, we observed that native urethral biopsies and the implanted mass-cultured urethral cells did not express uroplakin-3 in Swiss nu/nu mice, apart from the expression in one single “micro-bladder”-like structure, arising from a urethral holoclone. This lets us assume that urothelial umbrella cells arising from urethral holoclones can express uroplakin-3 if implanted underneath the kidney capsule, although it is not expressed in the native urethra. Indicating that urethral holoclones can under certain condition have a broaden differentiation capacity.

We also investigated if there is a difference in the behavior of urothelial cells harvested from the bladder dome or the trigone. As it has been reported that urothelial cancers often develops in the trigone, it could be speculated that the trigonal region has an increased stem cell pool and would therefore be optimal for cell harvesting [24]. However, we observed no difference in the cell growth and differentiation capacity of cells from the two harvest locations. These data correlate well with histological assessments of the human bladder, where clonal patches were found to replenish urothelium in all regions of the bladder, and not particularly from the trigone [25].

Porcine clonogenic urothelial cells seem to favor the 3T3-J2 culture system for continuous self-renewal, in contrary to human clonogenic urothelial cells. We can exclude a potential lentiviral bias for the human urothelial senescence since also non-transduced clonogenic human urothelial cells senesced. Thus, we observe an epithelial tissue difference between human keratinocytes and human urothelial cells, where *in vitro* cultured human clonal keratinocytes can reach 180 population doublings while clonal human urothelial cells can only reach 25 population doublings [26]. We argue that this is not only because of the 3T3-J2 culture system is optimized for keratinocyte growth, but that normal *in vivo* human urothelium cell turnover is also estimated to be slower (6–11 months), and inherently human urothelial cells should have a lower growth potential than the human keratinocytes (20 days epidermal cell turnover) [27], [28]. However, the 3T3-J2 culture system allows us, during a period of two weeks, to evaluate human urothelial cell self-renewal and to study which molecular signatures, such as p63, are important for self-renewal [29]. Since p63 has been reported in keratinocyte and cornea holoclones and more recently has been implicated as an *in vivo* signature of mouse bladder stem cells, we saw this as a likely molecular signature of human urothelial holoclones [5], [30], [31]. Proliferation data from holoclones showed that they senesced at a late passage and this correlated with a mixed and weak expression of p63 in the few occurring urothelial colonies. However, the urothelial holoclones at an early passage (in a self-renewing phase) expressed p63 homogenously and strongly in the urothelial colonies. We also observed that the clonal hierarchy reflected the level of p63 expression, where the holoclone had a more homogenous expression of p63 compared to the meroclone. It is tempting to infer from the biopsy data of p63 expression in native human ureter, where the p63 expression from basal to superficial cell goes from high to none, that the urothelial holoclones are derived from the basal layer of the urothelium and that the *in vitro* 3T3-J2 culture system reflects what we partially observe *in vivo*, similar to what has been described for skin model system [30]. We believe that further investigation in understanding the molecular mechanism for continuous human urothelial self-renewal is of importance to learn to retain stemness in long-term cell culture, which could be useful for potential clinical regenerative medicine applications.

We further plan to implant clonal cell populations into an orthotopic bladder model to determine if they can induce long-term functional reconstitution. Due to ethical concerns, clinical trials must be preceded by porcine functional reconstitution studies. Our finding that clonogenic porcine urothelial cells can be captured *in vitro* will allow us to perform long-term functional reconstitution experiments to determine how these single cell populations behave.

To date, only epidermal, corneal and blood-derived adult stem cell populations have been successfully used in long-term functional reconstitution for the treatment of severe diseases in human patients [1], [2], [3]. Our results suggest that clonogenic urothelial stem cell populations could potentially represent an additional adult stem cell population, to be used for functional regeneration of the diseased human urinary tract.

## Conclusions

Human clonogenic urothelial stem cells have not previously been studied *in vitro* or *in vivo*. We showed that isolated clonogenic, human ureteral urothelial cells are able to self-renew *in vitro* and to fully differentiate *in vivo*. Future larger-animal functional reconstitution studies of *in vitro* cultured clonogenic urothelial stem cells will be useful before initiating clinical trials in human patients. Toward this end, we showed that porcine clonogenic urothelial stem cells exist and therefore can be used for such studies.

## Supporting Information

Figure S1 **Colony forming capacity of mass-cultured human and porcine urothelial** **cells.** (A) Isolated human ureteral cells, (B) porcine ureteral cells, (C) porcine urethral cells, (D and E) porcine bladder dome and trigonal cells.
(TIF)

Figure S2 ***In vitro*** **and** ***in vivo*** **behavior of mass-cultured human ureteral cells.** (A and B) Growth curves and colony forming capacity of isolated human ureteral urothelial cells. (C) Cytokeratin 7, uroplakin-2 and uroplakin-3 expression in native human ureteral tissue. (D) Cytokeratin 7, uroplakin-2 and uroplakin-3 expression of *in vitro* cultured human ureteral urothelial cells after 8 days. (E) Cytokeratin 7, uroplakin-2 and uroplakin-3 expression of *in vivo* implanted human ureteral urothelial cells after 3 wk (scale bars, 20 µm).
(TIF)

Figure S3 ***In vitro*** **and** ***in vivo*** **behavior of mass-cultured porcine ureteral cells.** (A and B) Growth curves and colony forming capacity of isolated porcine ureteral urothelial cells. (C) Cytokeratin 7, uroplakin-2 and uroplakin-3 expression in native porcine ureteral tissue. (D) Cytokeratin 7, uroplakin-2 and uroplakin-3 expression of *in vitro* cultured porcine ureteral urothelial cells after 8 days. (E) Cytokeratin 7, uroplakin-2 and uroplakin-3 expression of *in vivo* implanted porcine ureteral urothelial cells after 3 wk (scale bars, 20 µm).
(TIF)

Figure S4 ***In vitro*** **and** ***in vivo*** **behavior of mass-cultured porcine bladder dome cells.** (A and B) Growth curves and colony forming capacity of isolated porcine bladder dome urothelial cells. (C) Cytokeratin 7, uroplakin-2 and uroplakin-3 expression in native porcine bladder dome tissue. (D) Cytokeratin 7, uroplakin-2 and uroplakin-3 expression of *in vitro* cultured porcine bladder dome urothelial cells after 8 days. (E) Cytokeratin 7, uroplakin-2 and uroplakin-3 expression of *in vivo* implanted porcine bladder dome urothelial cells after 3 wk (scale bars, 20 µm).
(TIF)

Figure S5 ***In vitro*** **and** ***in vivo*** **behavior of mass-cultured porcine bladder trigone cells.** (A and B) Growth curves and colony forming capacity of isolated porcine bladder trigone urothelial cells. (C) Cytokeratin 7, uroplakin-2 and uroplakin-3 expression in native porcine bladder trigone tissue. (D) Cytokeratin 7, uroplakin-2 and uroplakin-3 expression of *in vitro* cultured porcine bladder trigone urothelial cells after 8 days. (E) Cytokeratin 7, uroplakin-2 and uroplakin-3 expression of *in vivo* implanted porcine bladder trigone urothelial cells after 3 wk (scale bars, 20 µm).
(TIF)

Figure S6 ***In vitro*** **and** ***in vivo*** **behavior of mass-cultured porcine urethral cells.** (A and B) Growth curves and colony forming capacity of isolated porcine urethral urothelial cells. (C) Cytokeratin 7, uroplakin-2 and uroplakin-3 expression in native porcine urethral tissue. (D) Cytokeratin 7, uroplakin-2 and uroplakin-3 expression of *in vitro* cultured porcine urethral urothelial cells after 8 days. (E) Cytokeratin 7, uroplakin-2 and uroplakin-3 expression of *in vivo* implanted porcine urethral urothelial cells after 3 wk (scale bars, 20 µm).
(TIF)

Figure S7 **Back skin model for** ***in vivo*** **urothelial differentiation.** (A and B) Hematoxylin & eosin (H&E) staining of an implanted urothelial sheet into the dorsal subdermal space of Swiss nu/nu mice (A: scale bar 500 µm, B: scale bar 50 µm). (C and D) Immunohistochemistry of an implanted urothelial sheet using antibodies against uroplakin-2 and uroplakin-3. Note no uroplakin-3 expression (D).
(TIF)

Figure S8 **Immunohistochemistry of skin and thymus acting as negative control.** (A) Cytokeratin 7, uroplakin-2 and uroplakin-3 expression in native porcine skin tissue. (B) Cytokeratin 7, uroplakin-2 and uroplakin-3 expression of *in vitro* cultured porcine keratinocytes after 8 days. (C) Cytokeratin 7, uroplakin-2 and uroplakin-3 expression of *in vitro* cultured porcine epithelial thymus epithelial cells after 8 days.
(TIF)

Table S1 **Tissue donor information.** (A) Human donor information. (B) Porcine donor information.
(TIF)
