# Supplementary figures and images for: Clonal, Self-Renewing and Differentiating Human and Porcine Urothelial Cells, a Novel Stem Cell Population
Source: PLoS One. 2014 Feb 26;9(2):e90006. doi: 10.1371/journal.pone.0090006 (PMC3935977; doi:10.1371/journal.pone.0090006)

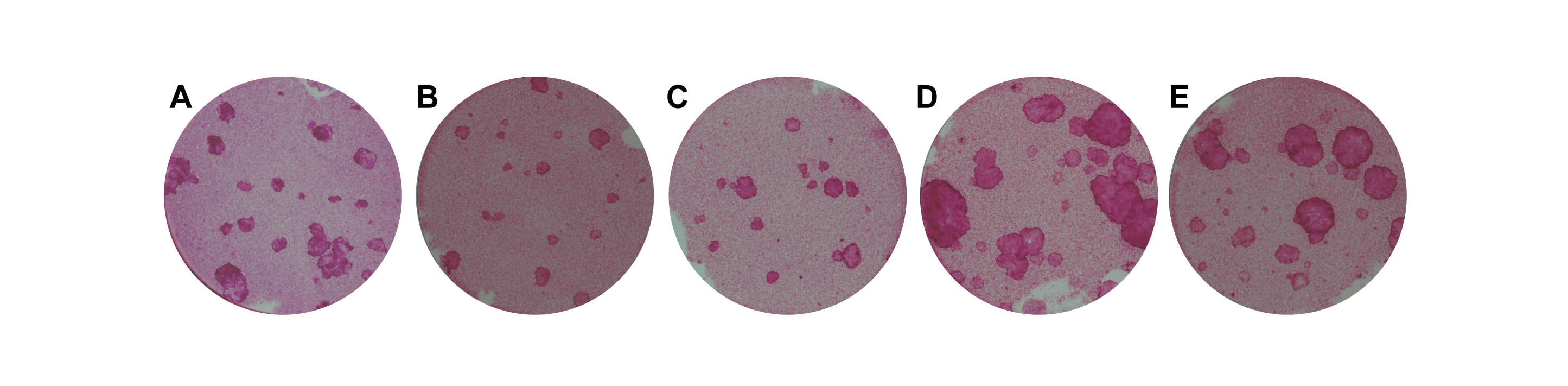

Supplement: Figure S1 — Colony forming capacity of mass-cultured human and porcine urothelial cells. (A) Isolated human ureteral cells, (B) porcine ureteral cells, (C) porcine urethral cells, (D and E) porcine bladder dome and trigonal cells. (TIF) [file pone.0090006.s001.tif]

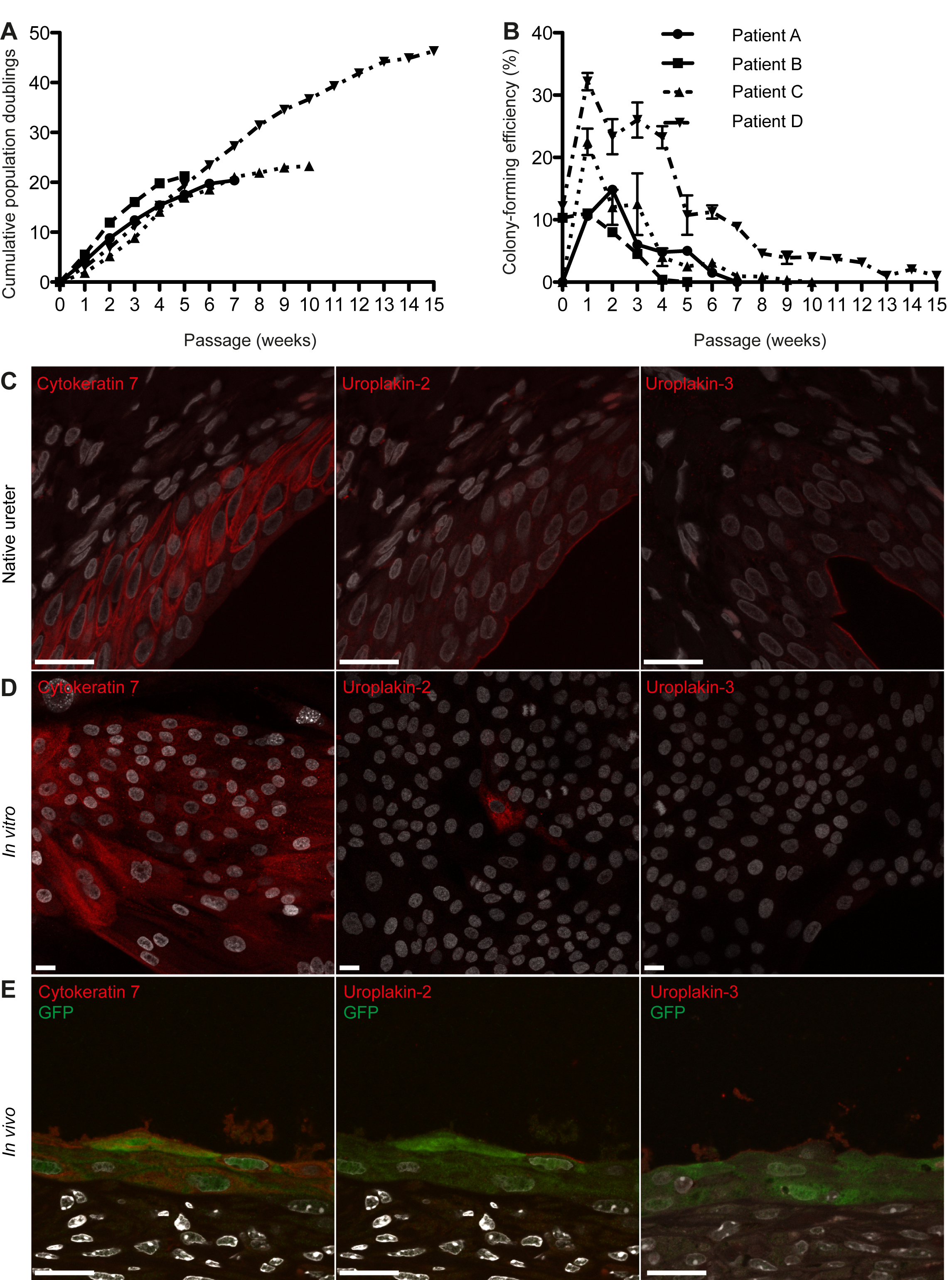

Supplement: Figure S2 — In vitro and in vivo behavior of mass-cultured human ureteral cells. (A and B) Growth curves and colony forming capacity of isolated human ureteral urothelial cells. (C) Cytokeratin 7, uroplakin-2 and uroplakin-3 expression in native human ureteral tissue. (D) Cytokeratin 7, uroplakin-2 and uroplakin-3 expression of in vitro cultured human ureteral urothelial cells after 8 days. (E) Cytokeratin 7, uroplakin-2 and uroplakin-3 expression of in vivo implanted human ureteral urothelial cells after 3 wk (scale bars, 20 µm). (TIF) [file pone.0090006.s002.tif]

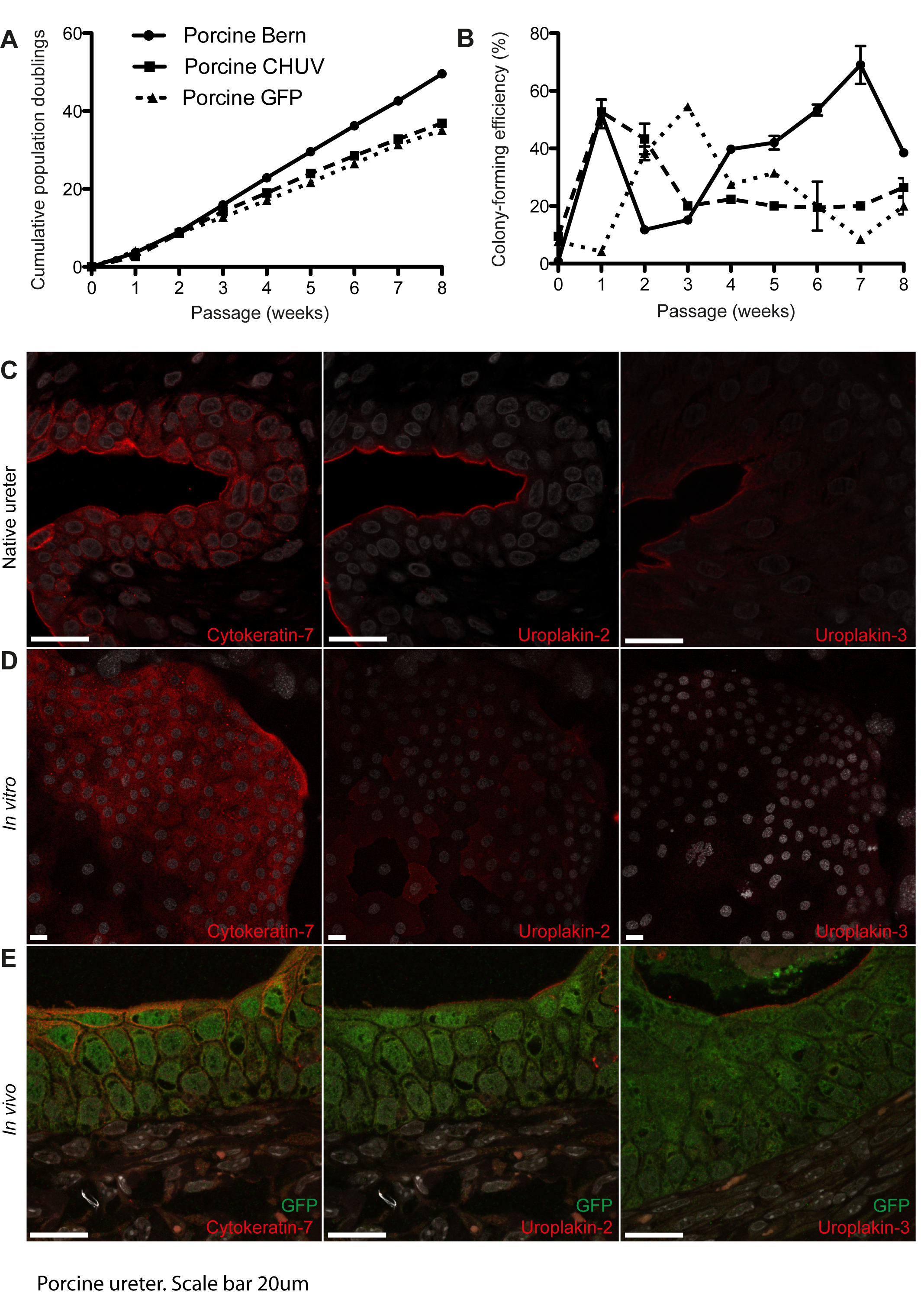

Supplement: Figure S3 — In vitro and in vivo behavior of mass-cultured porcine ureteral cells. (A and B) Growth curves and colony forming capacity of isolated porcine ureteral urothelial cells. (C) Cytokeratin 7, uroplakin-2 and uroplakin-3 expression in native porcine ureteral tissue. (D) Cytokeratin 7, uroplakin-2 and uroplakin-3 expression of in vitro cultured porcine ureteral urothelial cells after 8 days. (E) Cytokeratin 7, uroplakin-2 and uroplakin-3 expression of in vivo implanted porcine ureteral urothelial cells after 3 wk (scale bars, 20 µm). (TIF) [file pone.0090006.s003.tif]

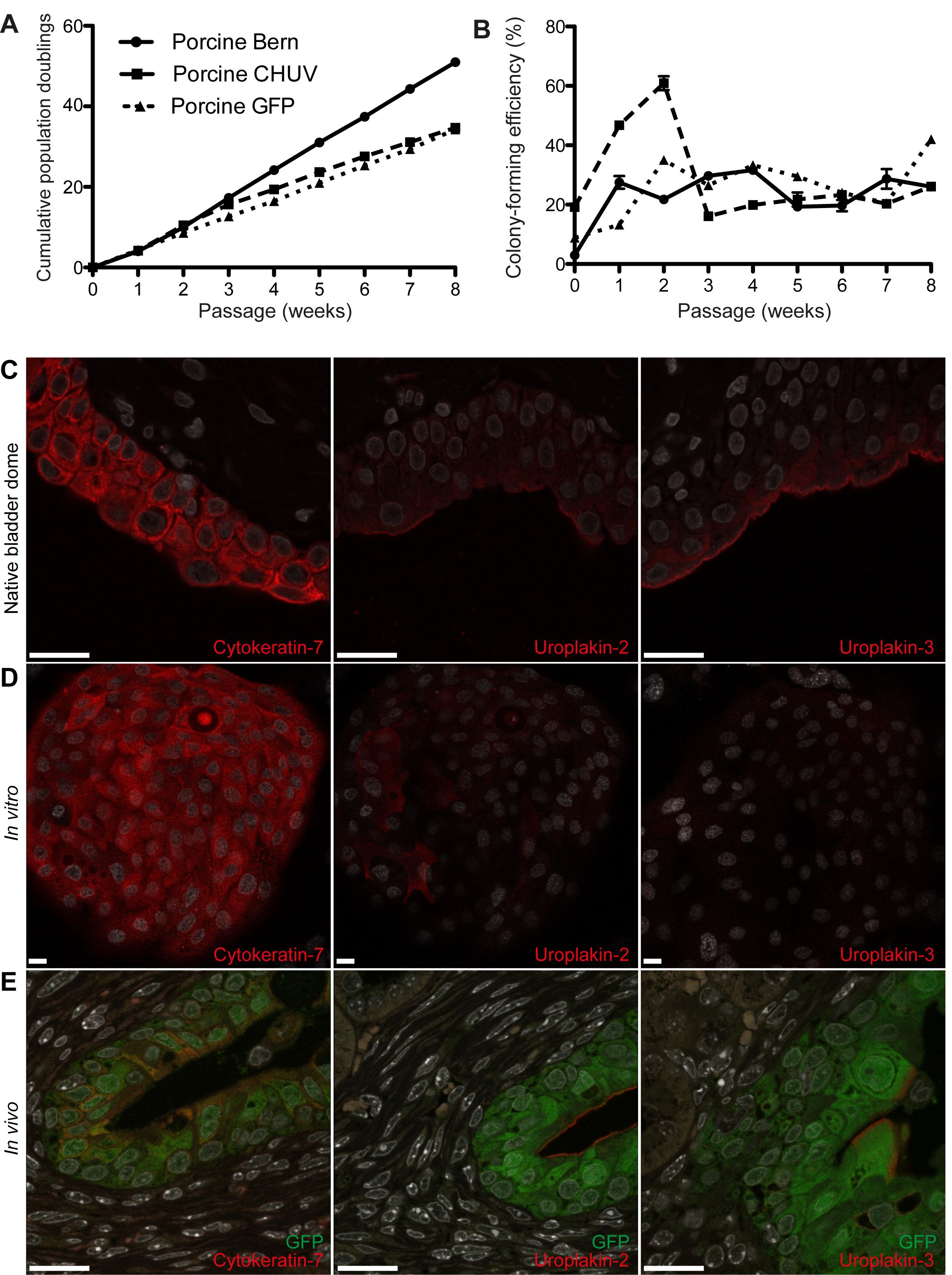

Supplement: Figure S4 — In vitro and in vivo behavior of mass-cultured porcine bladder dome cells. (A and B) Growth curves and colony forming capacity of isolated porcine bladder dome urothelial cells. (C) Cytokeratin 7, uroplakin-2 and uroplakin-3 expression in native porcine bladder dome tissue. (D) Cytokeratin 7, uroplakin-2 and uroplakin-3 expression of in vitro cultured porcine bladder dome urothelial cells after 8 days. (E) Cytokeratin 7, uroplakin-2 and uroplakin-3 expression of in vivo implanted porcine bladder dome urothelial cells after 3 wk (scale bars, 20 µm). (TIF) [file pone.0090006.s004.tif]

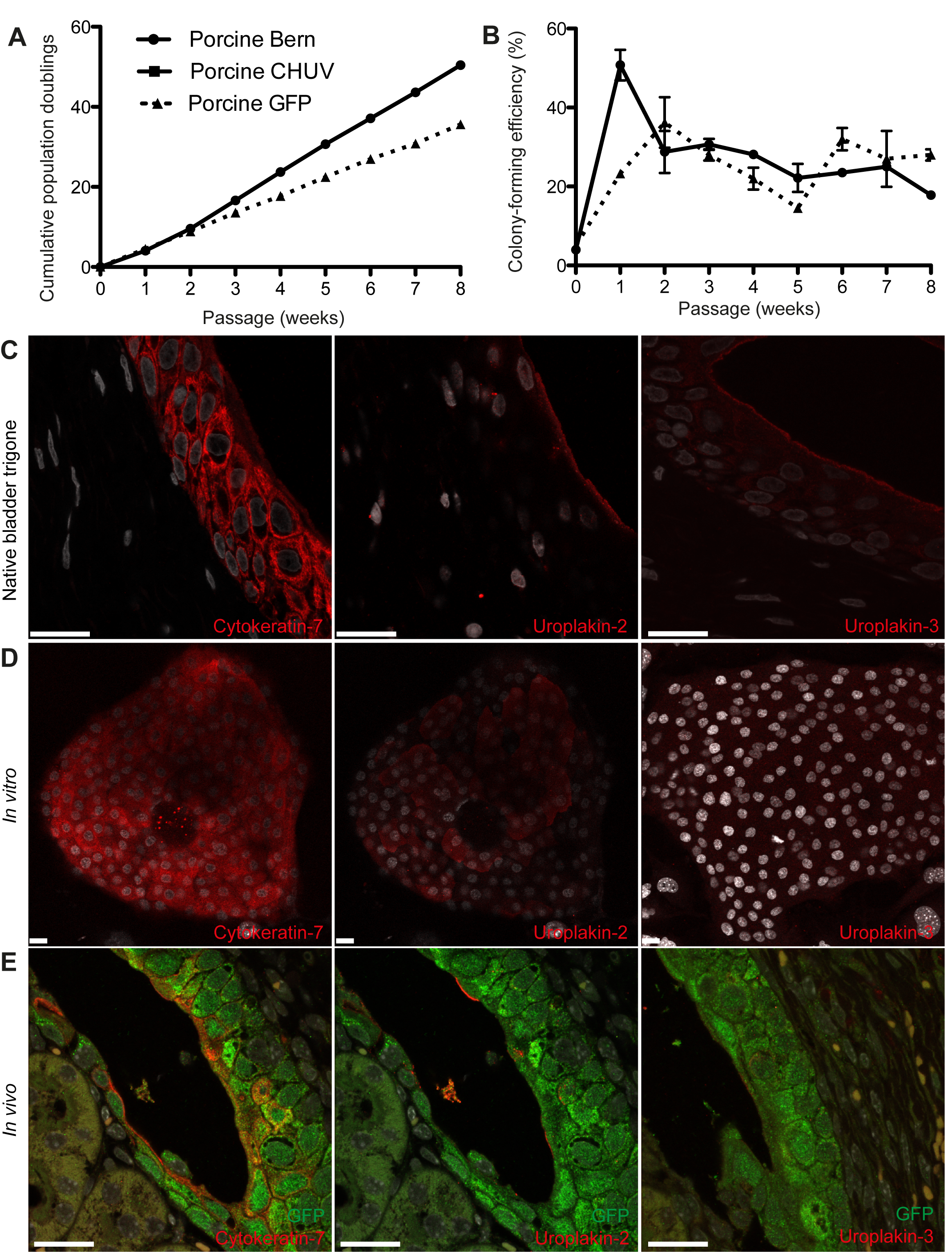

Supplement: Figure S5 — In vitro and in vivo behavior of mass-cultured porcine bladder trigone cells. (A and B) Growth curves and colony forming capacity of isolated porcine bladder trigone urothelial cells. (C) Cytokeratin 7, uroplakin-2 and uroplakin-3 expression in native porcine bladder trigone tissue. (D) Cytokeratin 7, uroplakin-2 and uroplakin-3 expression of in vitro cultured porcine bladder trigone urothelial cells after 8 days. (E) Cytokeratin 7, uroplakin-2 and uroplakin-3 expression of in vivo implanted porcine bladder trigone urothelial cells after 3 wk (scale bars, 20 µm). (TIF) [file pone.0090006.s005.tif]

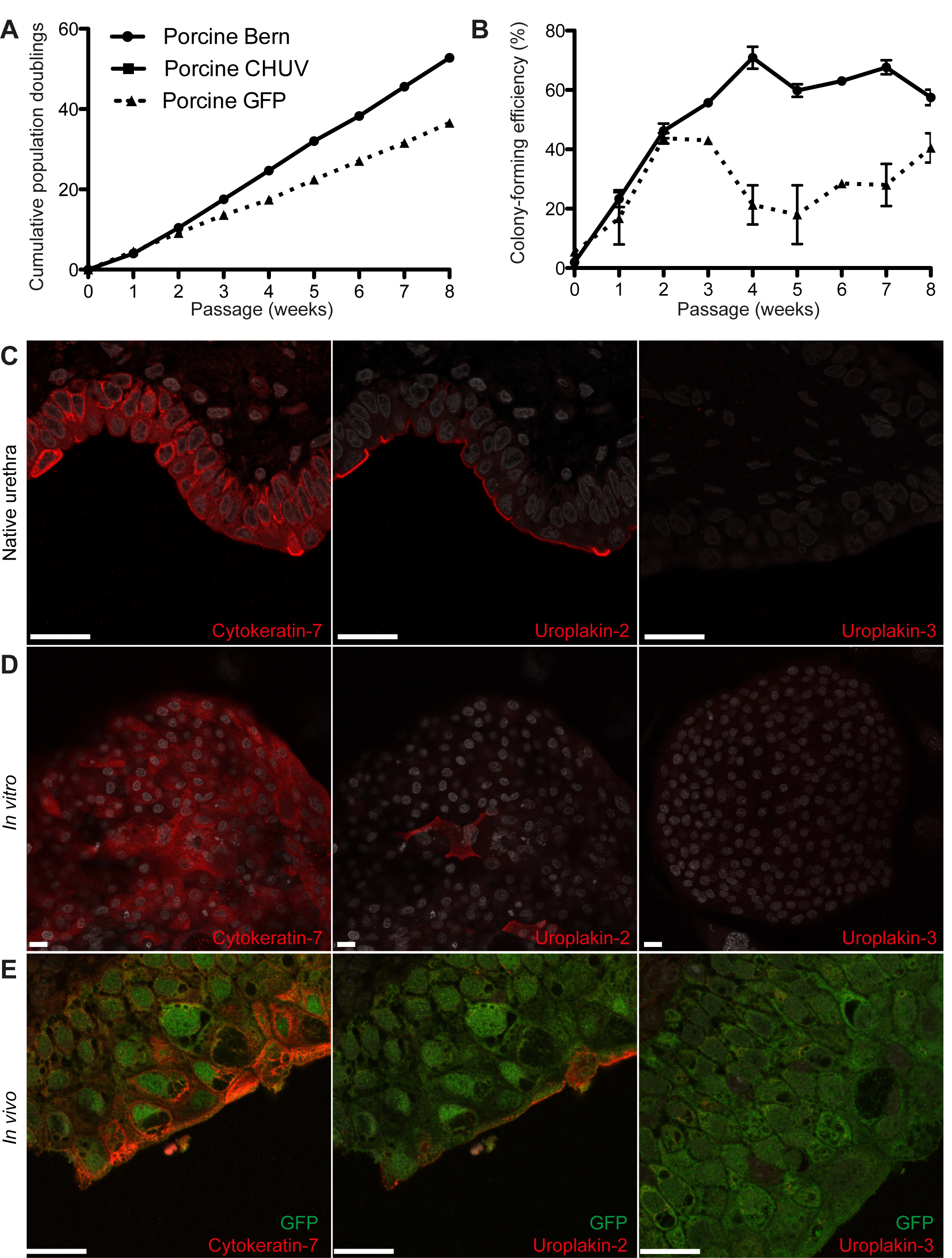

Supplement: Figure S6 — In vitro and in vivo behavior of mass-cultured porcine urethral cells. (A and B) Growth curves and colony forming capacity of isolated porcine urethral urothelial cells. (C) Cytokeratin 7, uroplakin-2 and uroplakin-3 expression in native porcine urethral tissue. (D) Cytokeratin 7, uroplakin-2 and uroplakin-3 expression of in vitro cultured porcine urethral urothelial cells after 8 days. (E) Cytokeratin 7, uroplakin-2 and uroplakin-3 expression of in vivo implanted porcine urethral urothelial cells after 3 wk (scale bars, 20 µm). (TIF) [file pone.0090006.s006.tif]

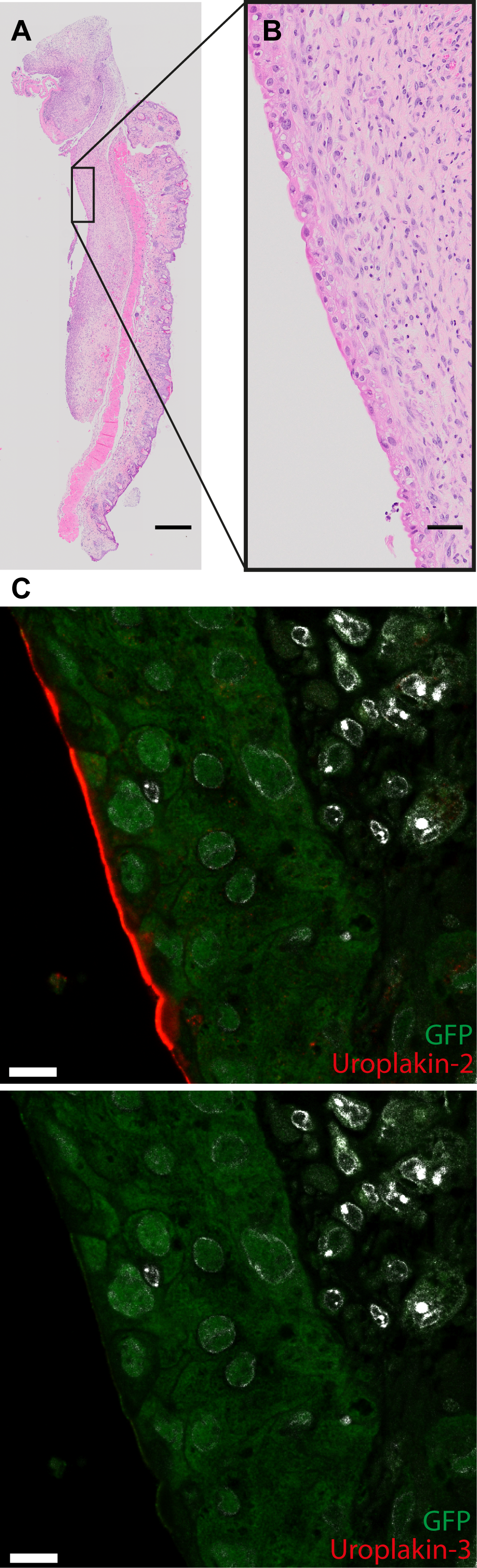

Supplement: Figure S7 — Back skin model for in vivo urothelial differentiation. (A and B) Hematoxylin & eosin (H&E) staining of an implanted urothelial sheet into the dorsal subdermal space of Swiss nu/nu mice (A: scale bar 500 µm, B: scale bar 50 µm). (C and D) Immunohistochemistry of an implanted urothelial sheet using antibodies against uroplakin-2 and uroplakin-3. Note no uroplakin-3 expression (D). (TIF) [file pone.0090006.s007.tif]

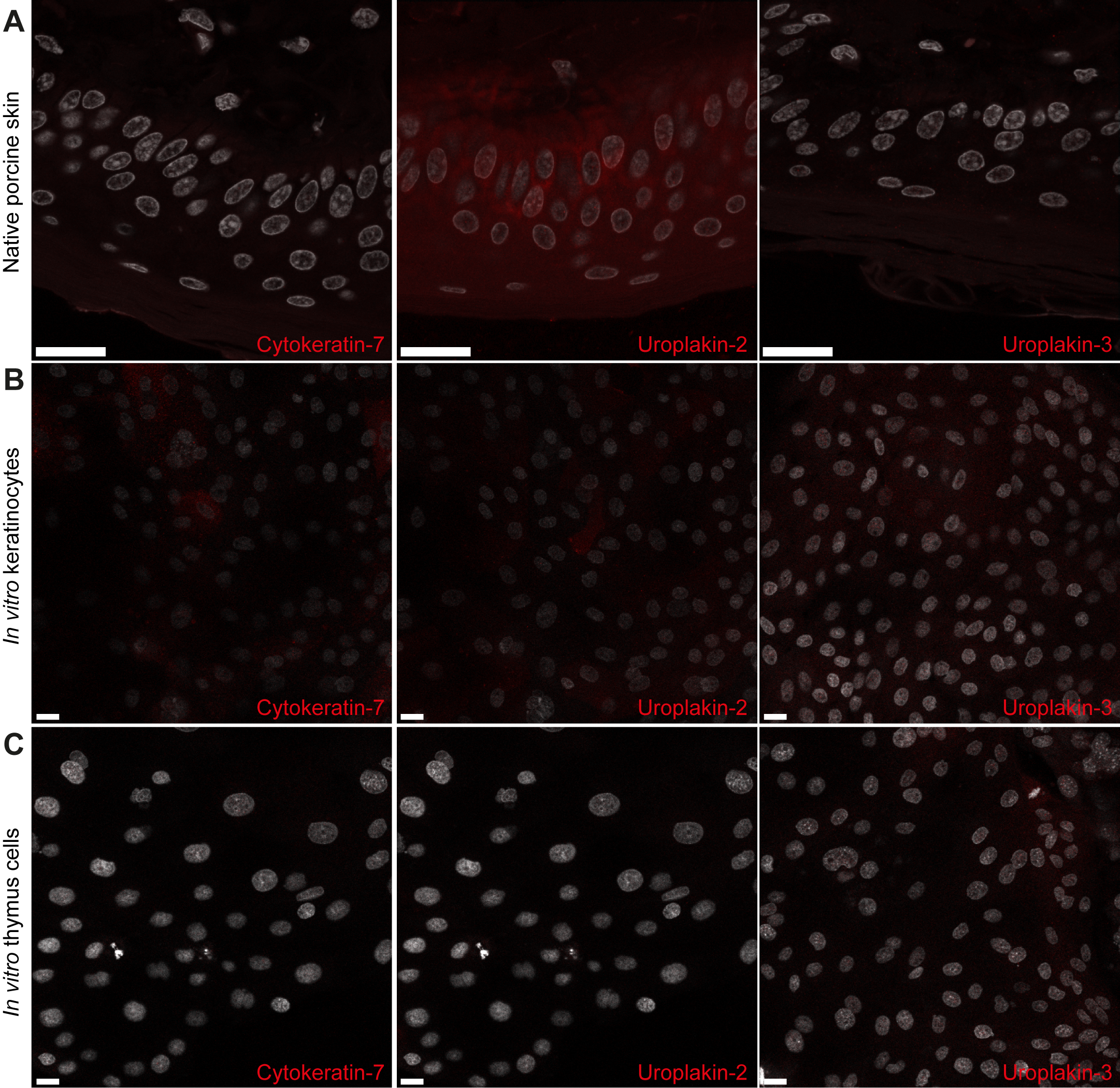

Supplement: Figure S8 — Immunohistochemistry of skin and thymus acting as negative control. (A) Cytokeratin 7, uroplakin-2 and uroplakin-3 expression in native porcine skin tissue. (B) Cytokeratin 7, uroplakin-2 and uroplakin-3 expression of in vitro cultured porcine keratinocytes after 8 days. (C) Cytokeratin 7, uroplakin-2 and uroplakin-3 expression of in vitro cultured porcine epithelial thymus epithelial cells after 8 days. (TIF) [file pone.0090006.s008.tif]

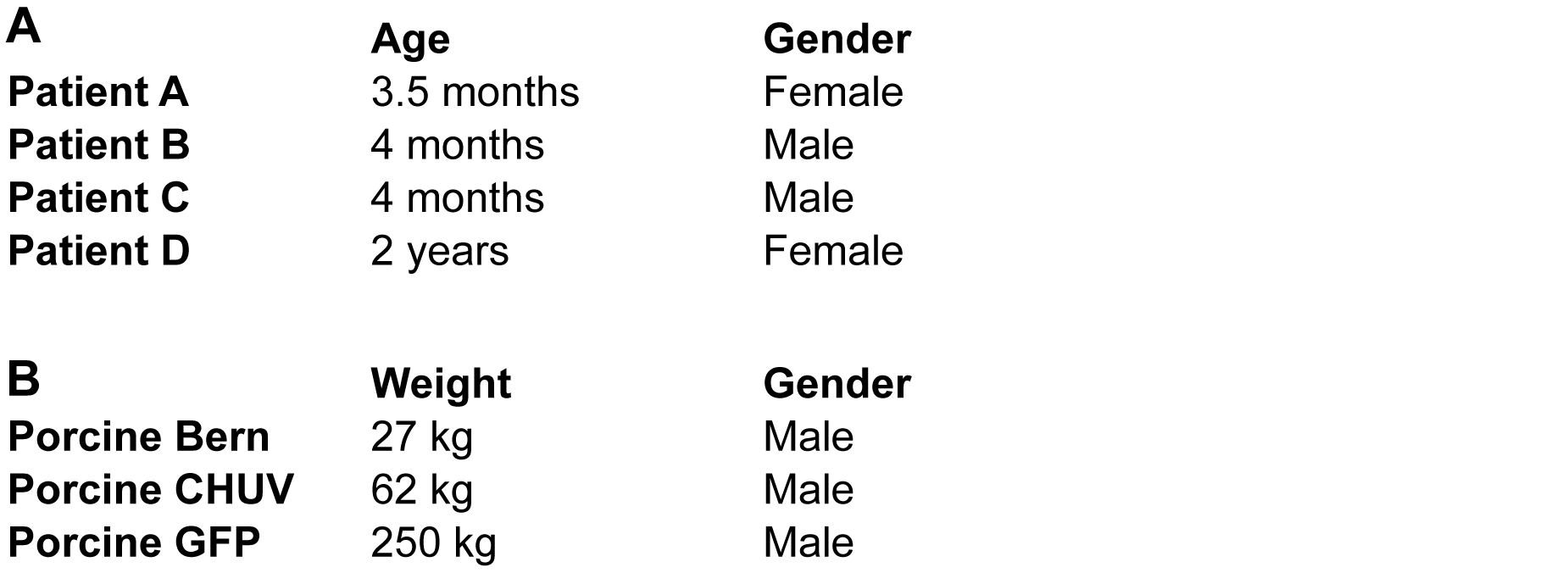

Supplement: Table S1 — Tissue donor information. (A) Human donor information. (B) Porcine donor information. (TIF) [file pone.0090006.s009.tif]
